# Modifications to advanced Core decompression for treatment of Avascular necrosis of the femoral head

**DOI:** 10.1186/s12891-017-1811-y

**Published:** 2017-11-21

**Authors:** Stefan Landgraeber, Sebastian Warwas, Tim Claßen, Marcus Jäger

**Affiliations:** Department of Orthopaedics and Trauma surgery, University Hospital of Essen, University of Duisburg-Essen, Hufelandstrasse 55, 45147 Essen, Germany

**Keywords:** Osteonecrosis, Hip, Core decompression, Bone graft substitutes, Tissue engineering

## Abstract

**Background:**

“Advanced Core Decompression” (ACD) is a new technique for treatment of osteonecrosis of the femoral head (ONFH) that includes removal of the necrotic tissue using a percutaneous expandable reamer followed by refilling of the drill hole and the defect with an injectable, hard-setting, composite calcium sulphate (CaSO_4_)-calcium phosphate (CaPO_4_) bone graft substitute. As autologous bone has been shown to be superior to all other types of bone grafts, the aim of the study is to present and evaluate a modified technique of ACD with impaction of autologous bone derived from the femoral neck into the necrotic defect.

**Methods:**

A cohort of patients with an average follow-up of 30.06 months (minimum 12 months) was evaluated for potential collapse of the femoral head and any reasons that led to replacement of the operated hip. Only patients in stages 2a to 2c according to the Steinberg classification were included in the study.

**Results:**

In 75.9% the treatment was successful with no collapse of the femoral head or conversion to a total hip replacement. Analysis of the results of the different subgroups showed that the success rate was 100% for stage 2a lesions and 84.6% respectively 61.5% for stages 2b and 2c lesions.

**Conclusions:**

Previous studies with a comparable follow-up reported less favourable results for ACD without autologous bone. Especially in stages 2b and 2c the additional use of autologous bone has a positive effect. In comparison to other hip-preserving techniques, the modified ACD technique is a very promising and minimally invasive method for treatment of ONFH.

**Trial registration:**

German clinical trials register (DRKS00011269, retrospectively registered).

## Background

Osteonecrosis of the femoral head (ONFH) is one of the main reasons for total hip arthroplasty (THA) in young patients [1]. A major disadvantage of hip replacement is that after a mean period of 15 to 25 years most implants become loose and need revision surgery [2]. This puts even young patients in a difficult situation as they can expect multiple revision surgeries during their lifetime with an increasing complication rate for each operation. Furthermore, the literature shows that the clinical outcome of primary hip arthroplasty in young patients has not improved over the last years. The mean Harris Hip Score for this group remains at 84.6 after a mean implantation time of 7.5 years [1]. Furthermore, they have a relatively high revision rate of 12.3%.

Therefore, other hip-preserving options for treating ONFH, apart from THA, are needed. “Advanced Core Decompression” (ACD) is a relatively new technique using a percutaneous expandable reamer that allows more efficient removal of necrotic tissue from the femoral head followed by refilling of the drill hole and the defect with an injectable, hard-setting, composite calcium sulphate (CaSO_4_)-calcium phosphate (CaPO_4_) bone graft substitute (PRO-DENSE®, Wright Medical Technology™, Inc. Arlington, TN, USA) [3–5]. Previous studies revealed a significant postoperative improvement in clinical and radiographic parameters in comparison to the preoperative situation and no surgery-related complications like fractures or infections were observed [3–5]. However, collapse of the femoral head still occurred depending on the follow-up period and stage of the femoral head defect. After a mean follow-up of 29 months, the results of ACD regarding the revision rate for conversion to a total hip replacement were nearly the same as for conventional core decompression [4]. One problem of the current technique is that PRO-DENSE®, the synthetic injectable bone graft used for this method, is only osteoconductive. An ideal bone graft should also have osteoinductive and osteogenetic characteristics [6, 7]. Therefore, Civinini et al. combined ACD with injection of concentrated autologous bone marrow aspirate (cBMA) to achieve osteoinduction [3]. However, this procedure does not induce osteogenesis. As autologous bone graft satisfies all the requirements for good bone remodelling in terms of osteoconduction, osteoinduction, osteogenesis as well as osteointegration, we intended to improve the surgical technique of ACD by combining it with autologous bone derived from the femoral neck.

The aim of the current paper is to describe and evaluate a modified surgical technique of ACD with the addition of autologous bone. A prospective study design was chosen. To make the results of the new technique comparable with the previous technique of ACD without the use of autologous bone, we carried out the evaluation after the same follow-up period chosen in a previous study [4]. As hip-preserving surgeries to treat ONFH are performed predominantly on defects classified as stage 2 according to the ARCO respectively the Steinberg classifications, we focused our evaluation of the new technique on that stage [8–10].

## Methods

### Surgical technique

**Fig. 1:**
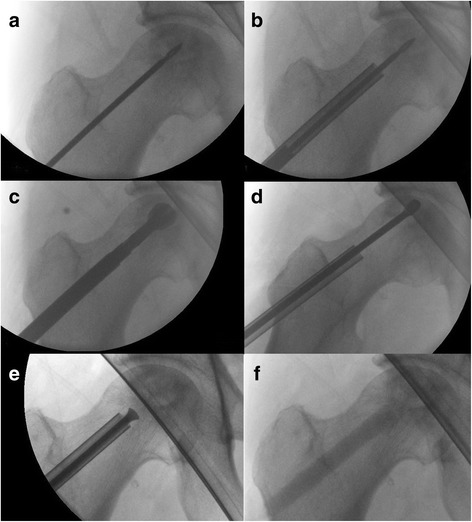
The main steps of the modified Advanced Core Decompression technique under fluoroscopic guidance: (**a**) A 3.2-mm diameter fluted guide wire is introduced close to the tuberculum innominati into the necrotic lesion. **b** Then a 9-mm cannulated drill bit is used to extend the diameter of the drill hole and a wire-guided trephine (see also Fig. 2) is used to harvest the cancellous bone from the femoral neck. **c** The next step is the introduction of the X-Ream^®^ expandable reamer into the drilling channel. This instrument is rotated and the blades expanded under fluoroscopic monitoring to debride as much of the dead bone as possible. **d** A sharp spoon is used for further debridement. **e** Finally, the core and the surgically created bone defect are backfilled first with autologous bone, (**f**) then with the PRO-DENSE^®^ injectable graft under fluoroscopic guidance

**Fig. 2:**
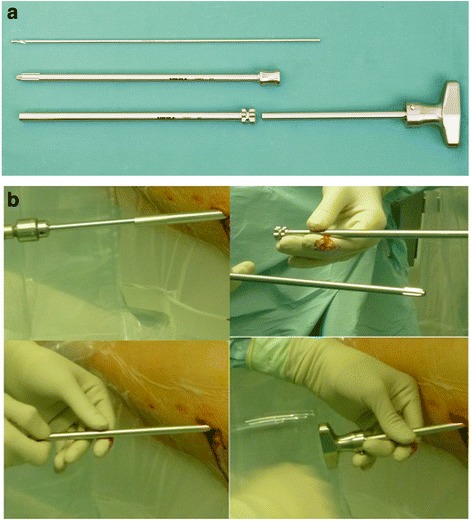
**a** The new tool for removal of autologous bone from the femoral head consists of the trephine with inboard cutting edges, the guiding device with a convex tip (introducer) and the handle containing a 3.2 mm canal for further guidance during the following introduction with hammer blows. **b** After cautious reaming to open the cortical bone, the trephine is introduced into the bone by means of a guidance device with a convex tip (introducer). When the trephine is in place, the introducer is removed and the proximal handle is attached. When the instrument has achieved its final position, it can be turned to cut respectively break the cylinder with the inside cutting edges

**Fig. 3:**
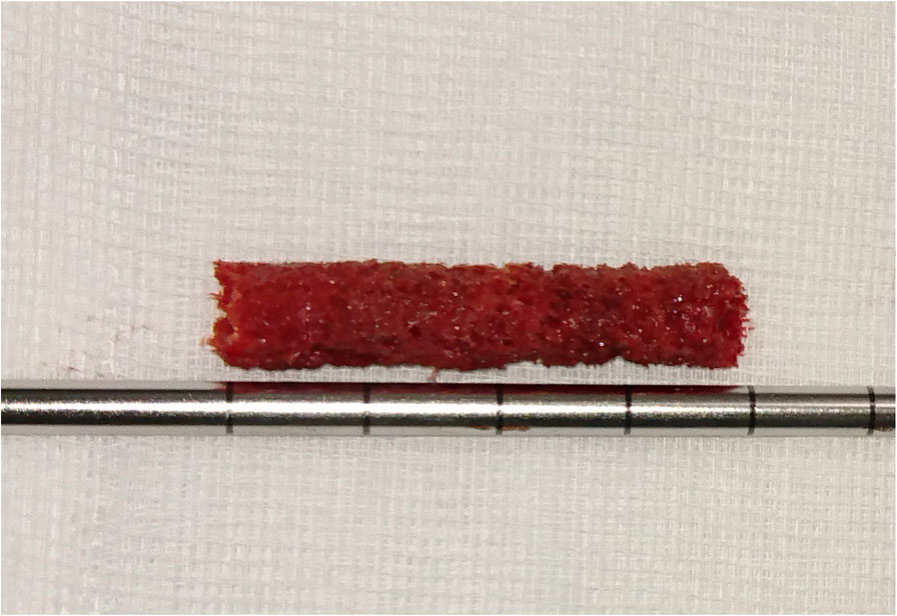
Typical cylinder of autologous bone derived from the femoral neck using the wire-guided trephine

The first steps of the procedure are identical to the ACD technique described in previous studies [3, 5]. Prior to surgery, preoperative planning was carried out using a digital planning tool (MediCad, HecTec GmbH, Landshut, Germany). The surgery is performed with the patient supine on a trauma table, which allows fluoroscopic guidance in the lateral and antero-posterior directions. After the optimal entrance point for drilling and a 1.5 cm skin incision near to the tuberculum innominatum has been found, a 3.2-mm diameter fluted guide wire is introduced into the necrotic lesion (Fig. 1a). Then a 9-mm cannulised drill bit is used to extend the diameter of the drill hole, but in contrast to the previous technique only to open up the cortical part of the femur. After removal of the 9-mm drill, the 3.2 mm guide wire remains in place. To harvest the cancellous bone from the femoral neck in the next step, a new type of trephine (Figs. 1b and 2) was specially developed for this indication in cooperation with a manufacturer for surgical devices (Eberle GmbH & Co. KG, Wurmberg, Germany). The introduction of the trephine into the bone is simplified by means of the conically shaped guidance device (introducer) that is guided by the guide wire. To facilitate a precise and reliable harvest of autologous bone and avoid deviation from the optimal core drilling, the handle of the trephine and in consequence the whole trephine is guided by the wire (Figs. 1b and 2). The trephine is then driven to the necrotic zone by well-dosed hammer blows. By turning the instrument, the inboard cutting edges at the instrument’s tip cut the cancellous bone and a bone cylinder can be removed. This step sometimes has to be performed twice in order to harvest as much cancellous bone as possible. Normally, a cylinder with a length of about 3.5 to 4 cm can be obtained (Fig. 3). Afterwards, the drilling is completed with the 9-mm cannulised drill under guidance of the guide wire. The surgeon must ensure under fluoroscopic guidance in both directions that the drill is placed in the best possible position without damaging the cartilage or cortical bone. A drilling in the centre of the necrotic area with a distance of about 3 to 5 mm to the subchondral, cortical surface is optimal in most cases. The next steps are once again the same as for the previous technique. The introduction of the working cannula is followed by introduction of the X-Ream^®^ expandable reamer (Wright Medical Technology Inc., Memphis, TN, USA) into the drilling channel (Fig. 1c). This instrument is rotated and the blades expanded under fluoroscopic monitoring to debride as much necrotic bone as possible. After removal of the X-Ream^®^, a sample of the necrotic tissue can be taken for pathological analysis to confirm the diagnosis. The remaining debrided tissue is removed by debriding with a sharp spoon (Fig. 1d) and flushing the drilling channel using a combination of irrigation and suction. These steps are important in order to remove the necrotic tissue as completely as possible and may be repeated several times to achieve better results. Differently from the previous surgical technique, the harvested cancellous bone is divided into small pieces. If autologous bone marrow aspirate (BMA) is used it should be added to the cancellous bone at this point. Whether BMA is used or not, the autologous bone is introduced through the working cannula into the reamed zone to fill the former necrotic defect as thoroughly as possible. A pestle from the advanced core decompression kit (Wright Medical Technology Inc., Memphis, TN, USA) is used to impact the autologous bone (Fig. 1e). The following steps are again the same as for the previous technique. The core and the surgically created bone defect are backfilled using the PRO-DENSE^®^ injectable (Wright Medical Technology Inc., Memphis, TN, USA) graft under fluoroscopic guidance (Fig. 1f). Discharge of PRO-DENSE^®^ into the soft tissue should be avoided, as CaPO_4_ may cause superficial soft tissue pain. The wound is closed in the standard fashion. After surgery, the patients rested in bed for 24 h until the PRO-DENSE^®^ had fully hardened, after which they were mobile with crutches for 4 weeks. Even though the use of PRO-DENSE^®^ allows full weight-bearing earlier, we prefer partial weight-bearing for a period of 4 weeks as we do not want to disturb ingrowth of the cancellous bone.

### Patients

This prospective study was approved by the local Ethics Committee (10–4293). Between August 2012 and March 2015, 31 hips of 25 patients were treated for ONFH in stage 2 according to the ARCO respectively Steinberg classifications by means of the modified ACD technique. Consequently, six patients were treated bilaterally with the modified ACD. Six further patients also had bilateral ONFH, but with a stage 3 defect on the contralateral side in three cases, and a stage 2 defect treated with the non-modified ACD also in three cases. One patient, who underwent modified ACD on both sides, also had osteonecrosis in both shoulders. The mean age of the 4 female and 21 male patients at the time of treatment was 42.9 years (21–60 years). Specific risk factors were evaluated in 21 cases. The reasons for ONFH were steroid use in eleven cases, steroid use in combination with chemotherapy in five, alcohol abuse in three, nicotine abuse (>30 cigarettes per day) in two and drug abuse in one case. In all the other cases ONFH was idiopathic.

All operations were performed by the first author in the Orthopaedic Clinic of the University of Duisburg-Essen and all patients were followed-up here.

Postoperatively, the patients took pain medication as required, and enoxaparine 40 mg for thrombosis prophylaxis for the period of partial weight-bearing. Additional medication that would influence the bone metabolism, like calcium or vitamin D, was not given.

The mean period up to the last follow-up examination was 30.06 months (12.00–43.30 months; SD: 10.11). Additionally, the patients were examined preoperatively and 6 weeks after surgery. Their medical histories, age, gender and risk factors were documented at the first visit and their modified Harris Hip Score (mHHS) and pain intensity, which was assessed by means of the visual analogue scale (VAS), at every visit. The mHHS is adapted from the HHS (physical range of motion measures removed) and can reach a maximum value of 91 [11].

The primary objective of the study was the evaluation of the survival rate of the femoral head. We defined failure of the surgery as the need for a hip replacement.

Evaluation of mHHS, VAS and survival rate of the femoral head were performed on basis of the Steinberg classification [9, 10]. To identify the respective stage of the ONFH, MRIs and X-rays were performed preoperatively for every patient. The MRIs were evaluated by two orthopaedic surgeons and at least one radiologist.

In addition, the Kerboul-combined necrotic angle was determined in preoperative MRIs [12] and the location of the osteonecrosis was categorized according to the a.p. view as peripheral or central.

Patients with signs of advanced osteoarthritis or ONFH in stages lower or higher than 2a to 2c according to the Steinberg classification were excluded from the study even if they underwent treatment with modified ACD. Postoperative radiographs were performed 1 day and 6 weeks after surgery. If collapse of the femoral head was suspected during follow-up, a further X-ray and if necessary an additional MRI were performed to evaluate possible progress of the necrosis.

### Statistical analysis

The Wilcoxon Test was used to compare the pre- and postoperative mHHS as well as the pre- and postoperative VAS (dependent samples). The Mann-Whitney U Test was used for comparison of the data of the individual subgroups 2a to 2c (independent samples). For comparisons of the pre- and postoperative results as well as for comparisons of the subgroups only the HHS and VAS of patients with a non-collapsed femoral head were considered.

The software SPSS 22 (IBM, Ehningen, Germany) was used to carry out the statistical computations.

## Results

One patient with a bilateral osteonecrosis died due to recurrent glioblastoma and was removed from the study. Up to the time of his death the patient did not have any complaints regarding his two operated hips. The remaining patients were available for follow-up.

No intraoperative or postoperative complications occurred in any patient. However, according to the definition of the primary objective of this study we observed treatment failure in 7 of 29 hips at the last follow-up examination and consequently a hip survival rate of 75.9%. All of these seven hips have been successfully revised and a total hip replacement implanted. Analysis of the survival rates of the femoral heads according to the stage of the disease showed that in cases with stage 2a ONFH the survival rate was 100% (3/3), in cases with stage 2b it was 84.6% (11/13) and in cases with stage 2c it was 61.5% (8/13).

The failure rate was higher in the bilateral group (5 of 11 patients, respectively 16 hips) than in the unilateral group (2 of 13 hips). But the osteonecrotic defects in the bilateral group also tended to be larger (2a: *N* = 1, 2b: *N* = 6, 2c: *N* = 9 (respectively 11 including the patient who died) in comparison to the unilateral group (2a: *N* = 2, 2b: *N* = 7, 2c: *N* = 4).

When treatment failures are viewed in relation to the Kerboul-combined necrotic angle, the mean Kerboul angle of the hips which were revised to a total hip replacement is slightly, but not significantly higher than that of those which did not require revision (207.4° vs. 199.0°). However, no collapse of the femoral head occurred in hips with a Kerboul angle of 160° or less.

In terms of location, a treatment failure was observed in two of six hips with a central lesion and five of 23 hips with a peripheral lesion. Separated for size, no treatment failure was observed in hips with a central located stage 2b osteonecrosis, but in two of 11 hips with a peripheral located stage 2b osteonecrosis. In hips with stage 2c osteonecrosis the failure rate was 50% (2 of 4 hips) if central located and 33,3% (3 of 9 hips) if peripheral located.

The age of the patients with treatment failure was higher than that of the patients without treatment failure (47.9 vs 41.8 years). But this difference was not significant.

In total, 14 hips were treated with autologous bone in combination with autologous bone-marrow aspirate (BMA) that was not subjected to substantial manipulation. Six of these cases were stage 2b ONFH, none of which showed treatment failure. The other cases were stage 2c ONFH. Of these, five hips (61.5%) were intact at the last follow-up, but three hips have since been revised to a total hip replacement.

The mean mHHS of all cases increased significantly (*p* < 0.001) from 73.65 points preoperatively to 86.27 points 6 weeks postoperatively. At the last follow-up, the mean mHHS of the remaining non-revised hips was 83.78 and consequently nearly at the same level. The mHHS for the different stages is given in Table 1. Statistical analysis showed no differences for the mHHS between the stages. The mean VAS of all cases decreased significantly (*p* < 0.001) from 3.61 preoperatively to 0.85 points 6 weeks postoperatively. At the last follow-up, the mean VAS of the remaining non-revised hips was 0.91 and thus also nearly at the same level. The VAS for the different stages is given in Table 1. Statistical analysis showed no differences for the VAS between the stages.

**Table 1:** Results of the pre- and postoperative examinations and the survival rate according to lesion size and stage (Steinberg classification).

Stage	Preoperative			6 weeks			Follow-up		
	N	mHHS	VAS	mHHS	VAS	Survival	mHHS	VAS	Survival
2a	3	82.00	3.67	91.00	0	100	89.67	0.33	100
2b	13	75.57	2.92	86.43	0.83	100	82.75	0.73	84.6
2c	13	69.93	4.23	85.00	1.08	84.6	83.13	1.38	61.5
2 (all)	29	73.65	3.61	86.27	0.85	100	83.78	0.91	75.9

(mHHS: Modified Harris Hip Score; VAS: Pain according to Visual Analogue Scale)

The trephine allowed harvesting of a 7-mm diameter bone cylinder in every patient. The mean length of the cylinder was 3.8 cm. The mean surgical time was 55 min.

## Discussion

With an overall success rate of 75.9% after a mean follow-up of 30.06 months the new ACD technique is a promising treatment option for stage 2 ONFH according to the ARCO respectively Steinberg classifications. After the same follow-up time, patients treated with the former ACD technique without autologous bone showed a hip survival rate of 67%, which was nearly the same as the survival rate of 65% reported for conventional core decompression [4, 13]. The improvement brought about by the modification is even more obvious when medium and larger-sized necrotic defects are compared. Here, the success rate increased from 71% to 84.6% for 2b defects and from 47.1 to 61.5% for 2c defects according to the Steinberg classification.

This improvement may be explained by the better biomechanical properties of autologous bone in comparison to PRO-DENSE^®^. It was shown by our group that the use of high-stiffness bone graft substitutes like PRO-DENSE^®^ results in critically high values of micromotion, normal stress and shear stress in the subchondral region [14]. The use of autologous bone allows refilling of the former necrotic area, especially in the subchondral region. For refilling of the remaining drilling canal PRO-DENSE^®^ bone graft has been shown to be useful, as it results in good bone remodelling when in contact with healthy bone and ensures stability of the femoral bone after ACD equal to that on the untreated opposite side [5, 15].

Besides biomechanical differences, autologous bone also has better biological properties than PRO-DENSE^®^, as it osteoconductive, osteoinductive and osteogenetic, whereas PRO-DENSE^®^ is only osteoconductive. In this context it is important to know that even people older than 25 years have blood-supplying bone marrow in the femoral neck [16]. The biological properties of the harvested autologous bone may be further improved by adding autologous bone marrow aspirate (BMA) from the iliac crest. Even if it was not the intention of the study to evaluate the effect of BMA that has not been subjected to substantial manipulation, our results show an additional positive effect in patients with a stage 2b ONFH. The study showed that patients with stage 2b ONFH who were treated by ACD in combination with autologous bone and BMA did not suffer from treatment failure. However, the positive influence of BMA was not observed in the large 2c defects, because of the eight patients who were treated additionally with BMA three later underwent revision for implantation of a total hip replacement, which means a survival rate of 62.5%. This is nearly the same survival rate of 60% that was observed for patients who underwent solely modified ACD without BMA. Larger defects, therefore, continue to prove difficult to treat, even if the results of modified ACD are better than those of the earlier ACD technique. A possible way of improving the survival rate of ACD of larger defects may be enhancement of the removal procedure of necrotic bone. Studies on the earlier ACD technique without the use of autologous bone showed that the initial as well as the remaining necrotic volume has an influence on the outcome [4, 17]. In.

In the current study it was observed that treatment failure did not appear in hips with a Kerboul-combined necrotic angle of 160° or less. This is a further indication that the lesion size seems to be important for the outcome, even if the Kerboul-combined necrotic angle in failed hips was not significantly larger than in the successfully treated hips. It is not possible to assess with any definite certainty if patients with bilateral osteonecrosis have a higher risk for failure. This group did have a higher failure rate, but the number of larger defects was also higher in this group. Treatment failure may also be associated with higher age, as the mean age of the failure group was 6.1 years older than the non-failure group. But this difference was not significant. To clarify if higher age is a predictor for treatment failure, a larger cohort of patients would be necessary. Also if the location of the osteonecrosis has an influence on the outcome cannot be conclusively clarified.

The usefulness of removing the necrotic bone and backfilling the defect with autologous bone in the treatment of ONFH has already been shown by other authors. Rijnen et al. used a larger approach, lifting the M. vastus lateralis and performing a 15 mm main drilling canal supplemented by multiple 9-mm drillings [18]. For ACD in contrast, a minimally invasive approach and a single 9-mm core drilling is performed. This is possible due to the use of the expandable X-ream. Furthermore, we consider that the use of the artificial bone graft (PRO-DENSE^®^) instead of allografts for backfilling of the femoral neck may be an advantage, as it is less expensive and safer. A further advantage of PRO-DENSE^®^ is the high stability of the femoral neck, which allows quick rehabilitation with full-weight bearing after two instead of 6 weeks. Furthermore, the mean surgical time of 55 min for the modified ACD was significantly shorter than the usual 90 min for the surgical technique of Rijnen et al. Comparison of the outcome of both methods for treatment of stage 2 ONFH after a similar follow-up period shows that modified ACD has a marginally better survival rate of 75.9% to 73%. Rijnen et al. evaluated a mean Harris-Hip-Score of 85. Given the fact that the mHHS has a 9-points lower maximum score than the HHS (91 to 100), the new technique of ACD presented here achieved considerably better results with a mean mHHS of 83.78. A comparison of defect sizes is not possible as these were not evaluated by Rijnen et al.

Another treatment option with removal of the necrotic tissue is the so-called trap-door or light-bulb procedure. With this method, the necrotic tissue is evacuated through a window made at the femoral head-neck junction and replaced by autologous bone or allograft. Various authors reported survival rates of the femoral head of 81% to 86% after long-term follow-up [19, 20]. Mont et al. also reported survival rates of 78.6% for large defects after a mean follow-up of 4 years [19]. In comparison to ACD, the trap door procedure is very invasive and more time-consuming. Furthermore, full weight-bearing is not allowed until after 3 months. Considering the higher morbidity of the trap-door procedure in comparison to modified ACD and the good outcome of stages 2a and 2b ONFH after treatment with modified ACD, we would prefer modified ACD for these patients. For stage 2c ONFH, further improvement of ACD with enhancement of the removal technique seems to be necessary to achieve satisfactory results.

For better comparability of the different methods further studies with similar study protocols and sufficient numbers of patients are needed.

## Conclusions

Even if it is still too early to draw final conclusions, the modified technique of ACD seems to be superior to the previous one. Further long-term follow-up is needed to receive final assurance. With the technique described in this paper, removal of necrotic tissue combined with refilling with autologous bone and additional injectable bone graft is easy to perform in a minimally invasive manner. Guidance of the trephine by the guide wire makes the harvesting of autologous bone from the femoral neck simple and sufficient. By harvesting adequate quantities of bone from the femoral neck it was possible to avoid the need to harvest additional autologous bone from the iliac crest and thus an increased risk of morbidity. A further advantage of the modification is that autologous bone is a good carrier for BMA and other cell-derived products.

## Abbreviations

ACD: Advanced Core Decompression
cBMA: Concentrated autologous bone marrow aspirate
HHS: Harris Hip Score
ONFH: Osteonecrosis of the femoral head
THA: Total hip arthroplasty
VAS: Visual analogue scale
